# Physical Health Monitoring for Children and Young People on Antipsychotic Medications in Salford CAMHS

**DOI:** 10.1192/bjo.2025.10594

**Published:** 2025-06-20

**Authors:** Pashyca Gill, Alastair Lawson, Hema Ramalingam

**Affiliations:** 1 Manchester University Foundation NHS Trust, Manchester, United Kingdom; 2 Salford CAMHS, Salford, United Kingdom

## Abstract

**Aims:** NICE guideline CG155 (Psychosis and Schizophrenia in children and young people: recognition and management) recommends that all children and young people who are commenced on an antipsychotic medication, should have physical health parameters (including height, weight, waist circumference, pulse rate, blood pressure, blood tests and ECG) completed at baseline, 12 weeks and 6 months. 100% compliance is expected.

Our aim was to review the current practice of physical health monitoring within Salford CAMHS, against these guidelines, and to improve compliance with these guidelines if found to be deficient.

**Methods:** Patients within the service who were prescribed antipsychotic medications on a single date (30/08/2023) were identified manually, by reviewing the caseloads of all prescribers of antipsychotic medication within the service. The electronic and paper notes of these patients were then reviewed, and a standardised proforma was used to collect data pertaining to the audit standards. The results were analysed and shared with the prescribers within the Salford CAMHS.

**Results:** The compliance for all the measured standards was below 50%. The physical health parameter completed most frequently was weight at baseline and 12 weeks; yet the compliance was only 47.8%. Blood tests and ECG were completed for 26% and 35% of patients, respectively, at baseline. 0% of the patients had waist circumference completed at any time point.

**Conclusion:** The results suggest that physical health monitoring within Salford CAMHS is non-compliant with NICE guidelines. However, the cases included in this audit ranged from 2015–2023, and given that only 9 of the 23 cases had been commenced on an antipsychotic within the last 12 months, the results may not reflect the practice of current prescribers in Salford CAMHS.

For 30% of cases, there was clinical documentation to suggest non-compliance with physical health monitoring due to mitigating complex needs. This included documentation of consent obtained from caregivers to continue without.

An action plan was devised including production of an antipsychotic monitoring checklist (with consent form), to be used by clinicians at baseline, 12 weeks and 6 monthly reviews. Discussions were held to identify a process for physical health parameters to be on a running tab in the electronic notes. In 12 months, following implementation of the action plan, there will be a re-audit of new cases. The re-audit will aim to clarify whether there has been an improvement in practice and should more accurately represent the current practice of prescribers in Salford CAMHS.

